# Geoepidemiology and clinical characteristics of neonatal lupus erythematosus: a systematic literature review of individual patients’ data

**DOI:** 10.3906/sag-1910-39

**Published:** 2020-04-09

**Authors:** Abdulsamet ERDEN, Antonis FANOURIAKIS, Levent KILIC, Alper SARI, Berkan ARMAĞAN, Emre BİLGİN, Yusuf Ziya SENER, Benazir HYMABACCUS, Fatih GÜRLER, Serdar CEYLAN, Sedat KİRAZ, Ömer KARADAĞ, Dimitrios T. BOUMPAS

**Affiliations:** 1 Division of Rheumatology, Department of Internal Medicine, Hacettepe University Faculty of Medicine, Ankara Turkey; 2 Rheumatology and Clinical Immunology, 4th Department of Internal Medicine, “Attikon” University Hospital, Athens Greece; 3 Department of Internal Medicine, Hacettepe University Faculty of Medicine, Ankara Turkey; 4 Joint Academic Rheumatology Program, School of Medicine, National and Kapodistrian University of Athens, Athens Greece

**Keywords:** neonatal lupus, congenital heart block, geoepidemiology

## Abstract

**Background/aim:**

Neonatal lupus erythematosus (NLE) is an autoimmune syndrome caused by transplacental transmission of maternal autoantibodies, often with devastating consequences. The objective of this systematic literature review was to analyze the demographic data, geoepidemiology, clinical, and serological characteristics associated with NLE.

**Materials and methods:**

We performed a systematic literature search of the Pubmed database covering the period from 1976 to August 2015, using the MeSH terms “neonatal lupus” or “congenital heart block”. To be included in the study, articles of any type (original articles, case series, and case reports) had to report on infants with NLE on an individualized (i.e. patient-by-patient) basis.

**Results:**

A total of 198 studies were included in the review, reporting on a total of 755 NLE patients. The most frequently reported clinical manifestations of NLE were congenital heart block (CHB, 65.2%), cutaneous lupus (33.1%), and cytopenias (15.5%). We found differences in NLE characteristics based on study geographical origin, with CHB being much more frequent in patients of European or American descent (49.4% and 35%, respectively), while reports originating from Asia reported a higher prevalence of skin involvement (45.2%). Most CHB cases (72.9%) were diagnosed between the 18th and 26th week of gestation.

**Conclusions:**

Phenotypic differences of NLE depending on race and country may reflect true pathophysiologic differences or methodologic discrepancies. While maternal autoimmune disease is not a prerequisite for the development of NLE, the existence of a truly “immunonegative” CHB is questionable.

## 1. Introduction

Neonatal lupus erythematosus (NLE) is an autoimmune syndrome caused by transplacental transmission of maternal antibodies, mainly anti SS-A/Ro and anti SS-B/La antibodies, and binding of these antibodies to foetal tissues [1,2]. Anti SS-A/Ro (60 kDa Ro, 52 kDa Ro or calreticulin) and anti SS-B/La are responsible for direct toxic effects on the myocardium and other organs, when transferred from mother to fetus [3,4]. The risk for congenital heart block (CHB) is ~ 1%–2%, in cases of maternal positivity for anti SS-A/Ro and/or anti SS-B/La antibody, but rises to almost 18% in cases with a history of CHB in a prior pregnancy (Brito-Zeron, Izmirly, Ramos-Casals, Buyon, & Khamashta, 2015) [5]. The presence of anti SS-A/Ro and anti SS-B/La antibodies is necessary but not sufficient to cause CHB and the complete NLE syndrome, as environmental and foetal factors seem to play an additional role [6–8]. Interestingly, at the time of NLE diagnosis, the majority of mothers do not have a prespecified rheumatic condition [9]. In the remaining patients, systemic lupus erythematosus (SLE), Sjögren’s syndrome, rheumatoid arthritis, and undifferentiated connective tissue diseases are most often seen [9,2]. 

Most data regarding NLE to date originate from observational studies and case reports/case series. Detailed data about the differences in geoepidemiology, frequency of organ manifestations other than CHB, situations that may be associated with recurrent NLE, and whether various therapeutic modalities affect the clinical outcomes, are scarce.

The objective of this systematic literature review was to analyse, on a patient-by-patient basis, the demographic data and geoepidemiology, maternal history traits, clinical and serological characteristics associated with NLE.

## 2. Materials and methods

We performed a systematic literature review of the Pubmed/MEDLINE database covering papers from studies published during the time period from 1976 to August 2015. Relevant publications were sought using the MeSH terms neonatal lupus or congenital heart block. Included publications were restricted to studies in humans and articles in English language. To be included in the review, articles of any type (original articles, case series and case reports) which reported on infants with NLE on an individualized *(*i.e. patient-by-patient) basis, for example giving information about the presence or absence of each component of NLE, were selected. Articles that did not include individual patient information (i.e. reported on frequency/percentage of characteristics in groups of patients) were excluded because this kind of information could not be subject to further comparative analyses. Review articles, editorials, randomized clinical trials, systematic reviews, and letters were also excluded. 

The initial selection process was based on titles and abstracts of retrieved articles, followed by full-text review of articles that were considered relevant. We documented the following variables: maternal age at NLE occurrence, connective tissue disease of the mother (if any) and disease duration, treatments received before and during pregnancy, gestational age at NLE diagnosis, organ manifestations of NLE, type and timing of CHB occurrence, treatments administered for CHB, type of delivery, and mortality. The literature search was performed by AE, article selection and data extraction were conducted by all authors collaboratively.

Because not every study reported all characteristics and outcomes of interest for this study, for each characteristic, a number in brackets is given (n=...), referring to the total number of patients for whom these data were available in the original studies.

The study protocol was approved by the local research ethics committee.

### 2.1. Statistical analysis

All statistical analyses were performed using SPSS software, version 21 (IBM Corp., Armonk, NY, USA). Continuous variables were tested for normality of data distribution, using the Kolmogorov–Smirnov test, and were expressed as mean (standard deviation, SD) and median (minimum-maximum, min-max) values, in case of normally and nonnormally distributed or ordinal data, respectively. Categorical variables were reported as frequencies (percentages) and were compared with the chi-square test. A P-value of <0.05 was considered significant for all comparisons.

## 3. Results

A total of 1125 abstracts of articles were evaluated. After excluding articles other than case reports/series and original articles and overlapping patients in case reports or original articles, the full text for 267 original articles/case reports were evaluated. Following full text evaluation, a total of 69 papers were excluded: 7 case reports and 15 original articles because of irrelevance, 10 case reports and 37 original articles because of providing insufficient data. A total of 198 studies were finally included in the analysis, reporting on a total of 755 NLE patients. A flow chart illustrating the systematic literature search for the identification of relevant studies is shown in Figure [details of the study designs for the studies included the review are provided in Table 1].

**Table 1:** Study designs of 198 studies recruited for review.

Article types	n (%)
Case series or case reports	147 (74.2%)
Prospective cohort studies	6 (3.0%)
Retrospective cohort studies	45 (22.8%)

### 3.1. Clinical manifestations of the NLE syndrome and differences in geoepidemiology

We identified a total of 755 cases of NLE from all of the included studies. At the time of NLE diagnosis, the mean (SD) age of mothers was 29.7 (4.8) years, and a little over one third (36%) were nulligravidae (Table 2). The most frequently reported clinical manifestations of NLE were CHB (492 patients, 65.2%), cutaneous lupus (250 patients, 33.1%), and cytopenias 117 (15.5%). Hepatic, pulmonary, and neurologic complications were less frequently reported (≤ 10% each) (Table 3). The majority of cases with NLE (any manifestation) were diagnosed postnatal (n = 357, 58%). However, in cases of cardiac involvement/CHB, the diagnosis was made during pregnancy in 72.1% (n = 256). Maternal age was not different between NLE cases with and without CHB [mean (SD) age 29.9 (4.4) vs. 29.0 (5.6) years, for cases with and without CHB development, respectively, P = 0.19).

**Figure 1:**
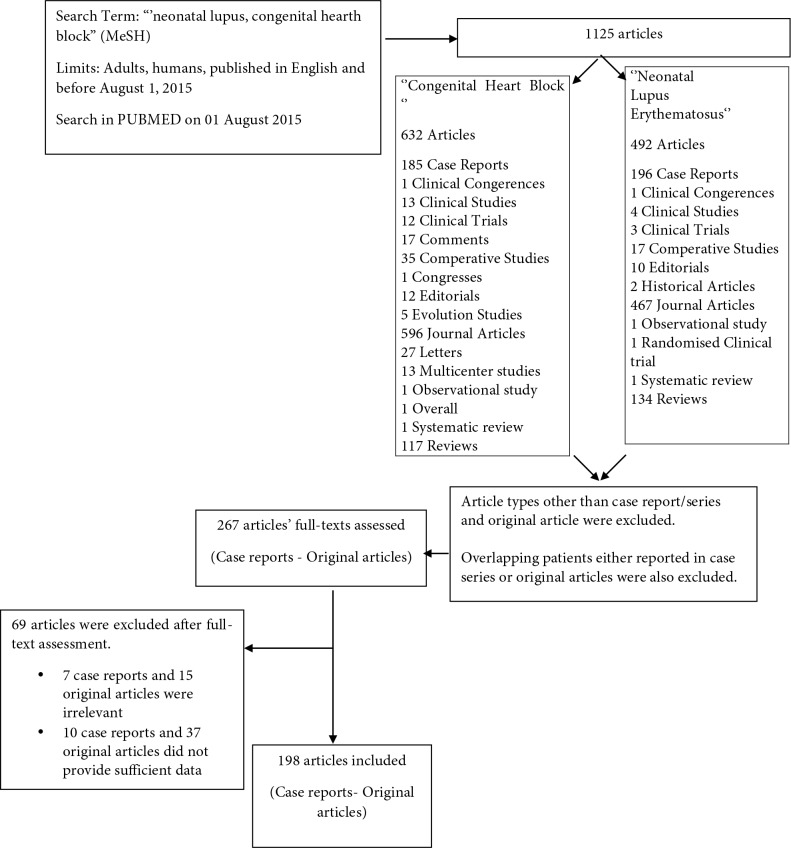
Flow chart demonstrating the article evaluation process for recruitment.

**Table 2:** Maternal characteristics of 755 patients with neonatal lupus.

Maternal characteristics	
Age in years, mean (SD)	29.72 (4.82)
Gestational week at CHB diagnosis, median (min-max)	23 (16–39)
Order of pregnancy, median (min-max)	2 (1–7)
N (%)^1^	
First	82 (36)
Second	94 (41.2)
Third	34 (15)
Fourth	13 (5.7)
≥Fifth	5 (2.1)
Maternal diagnosis, n (%)	
SLE	184 (24.4)
Sjögren’s syndrome	101 (13.4)
Undifferentiated connective tissue disease	53 (7.0)
Mixed connective tissue disease	9 (1.2)
Rheumatoid Arthritis	8 (1.1)
Leukocytoclastic vasculitis	3(0.4)
SLE+Antiphospholipid syndrome	3 (0.4)
Antiphospholipid syndrome	2 (0.3)
Systemic Sclerosis	1 (0.1)
SLE + Sjögren’s syndrome	1 (0.1)
Psoriasis	1 (0.1)
Other (hyperthyroidism, hypothyroidism, preeclampsia, Hashimoto, autoimmune hepatitis, hepatitis B, Graves’ disease, vitiligo)	20 (2.6)
None	188 (24.9)
Not specified	181 (24.0)
Diagnosis of autoimmune disease relative to pregnancy, n (%)^2^	
Before pregnancy	212 (64.8)
Postpartum period	97 (29.7)
During pregnancy	18 (5.5)
Maternal autoantibodies, n (%)	
Anti-SSA + anti-SSB	268 (35.5)
Anti-SSA	209 (27.7)
None (-)	41 (5.4)
ANA only	34 (4.5)
Anti-SSB	26 (3.4)
Anti-SSA+Anti-SSB+Anti-RNP	21 (2.8)
Anti-RNP	12 (1.6)
Anti-SSA+Anti-RNP	9 (1.2)
Not specified	135 (17.9)

^1^228 patients with known birth time were evaluated. ^2^Diagnosis time of collagen tissue disease were known for 327 mothers.

**Table 3:** General features of 755 patients with neonatal lupus erythematosus.

Manifestation, n (%)	
Heart block	492 (65.2)
Rash	250 (33.1)
Hematologic	117 (15.5)
Hepatic	78 (10.3)
Endocardial fibroelastosis	25 (3.3)
Pulmonary	10 (1.3)
Neurologic	9 (1.2)
Period of diagnosis, n (%)^1^	
Postpartum period	357 (58)
During pregnancy	258 (42)
Frequency of neonatal autoantibodies, n (%)	
Anti-SSA+Anti-SSB	111 (14.7)
Anti-SSA	106 (14)
Antibody (-)	20 (2.6)
Anti-RNP	17 (2.3)
Anti-SSB	10 (1.3)
Anti-SSA+anti-SSB+anti-RNP	5 (0.7)
Anti-SSA+anti-RNP	3 (0.4)
Not specified	483 (64)

^1^Period of diagnosis was calculated for 615 infants.

We observed differences in the frequency of the different clinical manifestations of the NLE syndrome, depending on the country of the origin of the original studies (Table 4). Interestingly, CHB was much more frequently reported in patients of European or American descent (49.4% (243/492) and 35% (172/492), respectively), while reports originating from countries of Asia reported a higher incidence of skin involvement (45.2% [113/250]) (Table 5).

**Table 4:** Clinical manifestations of NLE depending on geographic area of original studies and patients.

Continent of origin	Europe	America	Asia	P-value
Total number of cases, n	291	276	166	NA
CHB, n (%)	243 (83.5)	172 (62.3)	56 (33.7)	<0.001
Skin, n (%)	42 (22.0)	94 (34.0)	113 (68.0)	<0.001
Cytopenias, n (%)	19 (6.5)	54 (19.6)	43 (25.9)	<0.001
Hepatic, n (%)	19 (6.5)	43 (15.6)	16 (9.6)	0.002

**Table 5:** Distribution of patients with neonatal lupus erythematosus according to country and continent of origin.

Continent		Country		NLE (overall)1. Europe2. America3. Asia4. Australia5. Africa	n (%)291 (38.5) 276 (36.6) 166 (22.0)20 (2.6) 2 (0.3)
NLE (overall)1. USA2. Italy3. Sweden4. China5. Canada 6. Japan 7. Finland 8. Spain 9. India10. England 11. Australia12. Other	n (%)203 (26.9)81 (10.7)80 (10.6)70 (9.3)66 (8.7)56 (7.4)45 (6.0)29 (3.8)28 (3.7)24 (3.2)20 (2.6)53 (7.0)	CHB1. Europe2. America3. Asia4. Australia5. Africa	n (%)243 (49.4)172 (35)56 (11.4)20 (4.1)1 (0.2)
CHB1. USA2. Sweden 3. Italy4. Finland 5. India6. England7. Japan 8. Australia9. Spain 10. Canada11. Other	n (%)157 (31.9)71 (14.4)68 (13.8)44 (8.9)23 (4.7)22 (4.5)21 (4.3)20 (4.1)18 (3.7)15 (3.0)33 (6.7)	Cutaneous1. Asia2. America3. Europe4. Africa5. Australia	n (%)113 (45.2)94 (37.6)42 (16.8)1 (0.4)0 (0)
Cutaneous1. China2. USA3. Canada 4. Japan 5. Spain 6. Sweden7. France8. Taiwan9. Other	n (%)67 (26.8)51 (20.4)36 (14.4)35 (14.0)14 (5.6)8 (3.2)6 (2.4)5 (2.0)28 (11.2)	Hematologic1. America2. Asia3. Europe4. Africa 5. Australia	n (%) 54 (46.2)43 (36.8)19 (16.2)1 (0.9)0 (0)
Hematologic1. China2. Canada 3. USA4. Taiwan5. Spain 6. Sweden7. Other	n (%)33 (38.2)29 (24.8)21 (17.9)7 (6.0)5 (4.3)5 (4.3)17 (14.5)	Hepatic1. America2. Europe3. Asia	n (%) 43 (55.1)19 (24.4)16 (20.5)
Hepatic1. Canada 2. USA3. China4. Italy 5. Spain6. Other	n (%)23 (29.5)17 (21.8)8 (10.3)7 (9.0)5 (6.4)18 (23.0)	Pulmonary1. Europe2. America	n (%)7 (70)3 (30)
Pulmonary1. USA2. Italy 3. Spain 4. Rest of other countries	n (%) 3 (30)2 (20)2 (20)3 (30)

NLE: Neonatal lupus erythematosus; CHB: Congenital heart block.

In only 36% of cases (n = 272), the autoantibody status of infants was assessed by investigators (Table 3). Presence of anti-SSA, anti-SSB, and anti-RNP, either alone or in some combination thereof, were detected in 82.7%, 46.3%, and 9.1%, respectively; of note, in 20 cases (7.3%), no serum autoantibody could be detected. These seronegative infants did not differ significantly in terms of organ manifestations, week of diagnosis, survival rate or implantation of pacemaker from infants with autoantibodies.

### 3.2. Timing of CHB detection and which women are at risk? Maternal autoantibody status and disease in CHB

The status of maternal autoantibodies with CHB was reported in 376 mothers (76.4%) (Table 6). Anti SS-A/Ro, the most frequently detected autoantibody, was positive in 295 (78.4%) mothers; anti SS-B/La was positive in 181 (48.1%) mothers. Interestingly, 40 mothers (10.6% [40/376]) did not have any circulating autoantibodies. Accordingly, in 344 cases wherein information about maternal disease was available, the most frequent maternal diagnoses were SLE [101 (29.3%)] and Sjögren’s syndrome [75 (21.8%)], although other diseases were occasionally reported. No autoimmune rheumatic disease was evident in 110 cases (31.9%).

**Table 6:** Clinical characteristics of mothers and of infants with congenital heart block.

Maternal characteristics			Age in years, mean (SD)		29.9 (4.4)
Diagnosis of CHB, gestational week, mean (SD)		23.8 (4.5)	Male gender*, n (%)	
112 (45)	Birth weight (gr), mean (SD)-median (min-max)		2625 (745)–2690 (364–4370)
Delivery week, mean (SD)-median (min-max)		35.5 (4.4)–36 (19–43)	Pregnancy length^1^, n (%) Extreme preterm (≤25 weeks)Early preterm (26–32 weeks)Moderate preterm (32–34 weeks)Late premature (34–36 weeks)Term (37–42 weeks)Postmature (≥42 weeks)	
10 (4.9)17 (8.3)14 (6.8)67 (32.5)95 (46.1)3 (1.5)	Pregnancy order, n (%)^2^ First pregnancySecond pregnancyThird pregnancyFourth pregnancyFifth pregnancy and upper		44 (33.3)57 (43.2)22 (16.7)6 (4.5)3 (2.4)
Degree of CHB^3^, n (%)First Second Third (complete AV block)Sinus bradycardia		19 (6.4)27 (9.2)231 (78)19 (6.4)	Degree of CHB relativeto time of detection^4^, n (%)
Timing of CHB detection		During pregnancy		Postpartum period
First SecondThird (complete AV block)Sinus bradycardia	16 (84)18 (69)147 (70)15 (79)	3 (16)8 (31)60 (30)4 (21)
Timing of CHB diagnosis^5^, n (%) During pregnancy Postpartum period		256 (72.1)99 (27.9)	Maternal autoantibodies, n (%)Anti-SSA+Anti-SSBAnti-SSANoneANA onlyAnti-SSBAnti-SSA+Anti-SSB+Anti-RNP Anti-SSA+Anti-RNPAnti-RNPNot specified	
157 (31.9)124 (25.2)40 (8.1)25 (5.1)15 (3.0)9 (1.8)5 (1.0)1 (0.2)116 (23.6)	Foetal autoantibodies, n(%)Anti-SSAAnti-SSA+Anti-SSBNoneAnti-SSBAnti-RNPAnti-SSA+Anti-SSB+Anti-RNP Anti-SSA+Anti-RNPNot specified		49 (10)27 (5.5)7 (1.4)3 (0.6)2 (0.4)1 (0.2)1 (0.2)402 (81.7)

^1^Calculated over 206 patients with known pregnancy length. ^2^Calculated over 132 patients with known which ordered pregnancy she had a baby with NLE. ^3^Calculated over 296 patients with known type of CHB. ^4^Calculated over 271 patients with both known CHB type and diagnosis time. ^5^Calculated over 355 patients with known CHB diagnosis time. * Sex of 248 patients were known Gr: grammars; Min: minimum, Max: maximum, SD: standard deviation.

Mean (SD) gestational age at the time of detection in cases with CHB (n = 492 total) was 23.8 (4.5) weeks. Almost ¾ of cases (72.9%) with CHB were diagnosed between the 18th and 26th week of gestation. However, we found 4 cases (2.2%) diagnosed at weeks 16–17 and an additional 11.8% (n = 22), in which CHB was detected after 30th week of gestation (Table 7). Of note, less than half of pregnancies (46.1%, n = 95) resulted in a term pregnancy, while 20% resulted in at least moderate prematurity (≤34 weeks) (Table 8). The majority of cases had type III (i.e. complete) atrioventricular block (78.0%, n = 231).

**Table 7:** Distribution of gestational weeks for infants in whom congenital heart block was detected in utero.

Gestational week	n (%)
16	2 (1.1)
17	2 (1.1)
18	10 (5.3)
19	9 (4.8)
20	27 (14.4)
21	13 (6.9)
22	28 (14.9)
23	16 (8.5)
24	18 (9.6)
25	7 (3.7)
26	9 (4.8)
27	6 (3.2)
28	11 (5.9)
29	8 (4.3)
30	5 (2.7)
32	8 (4.3)
33	1 (0.5)
34	3 (1.6)
37	2 (1.1)
38	2 (1.1)
39	1 (0.5)
<18th week	4 (2.1)
18–26th week	137 (72.8)
27–34th week	42 (22.3)
>34th week	5 (2.7)
Total	188 (100)

**Table 8:** Pregnancy characteristics of foetuses with neonatal lupus erythematosus.

Male, n (%)	205 (43)
Birth weight (gr), mean (SD)	2650 (722)
Birth weight (gr), median (min-max)	2700 (364–4605)
Mode of delivery^1^, n (%)	
Caesarean section	123 (62.4)
Vaginal delivery	74 (37.6)
	
	
Way of conception^2^, n (%) SpontaneousIn vitro fertilization	182 (97.8)4 (2.2)
Delivery week, median (min-max)-mean (SD)	37 (19–43)–36.1 (4.1)
Pregnancy length^3^, n (%)	
Extreme preterm (≤25 weeks)	10 (3.3)
Early preterm (26–32 weeks)	17 (5.7)
Moderate preterm (32–34 weeks)	25 (8.4)
Late premature (34–36 weeks)	83 (28.0)
Term (37–42 weeks)	157 (53.0)
Postmature (≥42 weeks)	4 (1.3)

Min: minimum, Max: maximum, SD: standard deviation. ^1^Calculated from 197 patients with known way of birth. ^2^Calculated from 186 patients with known way of pregnancy. ^3^Calculated from 296 patients with known pregnancy length.

For 250 mothers, information on previous gestational history and birth outcome were provided by authors. Among them, 41 (16.4%) had given birth to a baby with NLE in a prior gestation. Recurrence rate of CHB was 31/250 (12.4%). All mothers with recurrent NLE were autoantibody positive. Interestingly, however, while the majority was positive for anti-Ro and/or anti-La (36/39, with available data), 3 women with recurrent NLE were negative for anti-Ro and anti-La and showed positivity only for ANA. Prevalence of rash was slightly higher in girls (55.8%) and those born at term (69.0%) (Table 9). 

**Table 9:** Assessment of NLE patients with rash.

Maternal characteristics	
Age in years, mean (SD)	28.7 (5.4)
Male/female*, n (%)	95 (44.2)/120 (55.8)
Birth weight (gr), mean (SD)	2723 (678)
Birth weight (gr), median (min-max)	2660 (1500–4605)
Delivery week, median (min-max)	38 (30–43)
Delivery week, mean (SD)	37.6 (2.7)
Distribution of pregnancy length^1^, n (%)	
Early preterm (26–32 weeks	2 (2.3)
Moderate preterm (32–34 weeks)	7 (8.3)
Late premature (34–36 weeks)	16 (19.0)
Term (37–42 weeks)	58 (69.0)
Post mature (42 weeks and upper)	1 (1.1)
Order of pregnancy, n (%)^2^	
First pregnancy	35 (34.6)
Second pregnancy	41 (40.5)
Third pregnancy	16 (15.8)
Fourth pregnancy	7 (6.9)
Fifth pregnancy	2 (1.9)
Distribution of maternal autoantibodies, n (%)	
Anti-SSA and Anti-SSB	85 (34)
Anti-SSA	56 (22.4)
Anti-RNP	16 (6.4)
Anti-SSB	7 (2.8)
Anti-SSA and Anti-SSB and Anti-RNP	3 (1.2)
Anti-SSA and Anti-RNP	2 (0.8)
Antibody (-)	11 (4.4)
Autoantibodies not known	70 (28)
Distribution of Fetal autoantibodies, n (%)	
Anti-SSA	
Anti-SSA and Anti-SSB	
None (-)	
Anti-SSB	
Anti-RNP	
Anti-SSA and anti-SSB and anti-RNP	
Anti-SSA and anti-RNP	
Not specified	

^1^Calculated over 84 patients with known pregnancy length. ^2^Calculated over 101 patients with known which ordered pregnancy she had a baby with NLE. * Sex of 215 patients were known Gr: gram, Min: minimum, Max: maximum, SD: standard deviation

## 4. Discussion

We have performed a systematic literature review to characterize the clinical spectrum of the NLE syndrome. Of note, our work focused on studies that provided detailed individual case presentations to understand better maternal and foetal characteristics (issues regarding therapy of NLE are the topic of a separate systematic review). CHB was the most prominent clinical manifestation (65.2% of cases), followed by cutaneous rash and cytopenias (hepatic, pulmonary, and neurologic complications were much less frequent). Presence of a maternal autoimmune disease was not a prerequisite for NLE, as in almost 1 in 3 pregnancies (31.9%) resulting in NLE, no disease was evident in the mother, Also, while anti-SSA/Ro was expectedly the most frequent autoantibody found in maternal serum, it is noteworthy that, in 10% of mothers (and 7.3% of neonates, accordingly), no circulating autoantibody could be detected in the serum; this observation clearly poses questions regarding the underlying pathophysiology of CHB in these cases. 

We separated all NLE cases depending on the country of origin of the study, and respective ethnic and racial distribution of cases, to identify potential differences in disease phenotype. Strikingly, we found that studies originating from Europe or America (mainly the the United States of America) reported significantly higher rates of CHB, the most serious complication of NLE (the risk of mortality ranges between 15% and 20%[10,11]), while studies in Asian patients revealed cardiac abnormalities in little over 30% of patients. On the contrary, cutaneous involvement was twice as frequent in studies from Asian countries versus other populations; as an illustrative example, in 2 studies from Japan and China, more than 80% of the combined 316 patients had cutaneous NLE, while the rate of CHB was 8.9%–23% [12,13]. While it is tempting to speculate about the involvement of genetic or environmental factors underlying this disparity, one must also be aware of the possibility of under-reporting, as well as the absence of a common definition, especially for CHB.

The notion that NLE is a syndrome mediated by the passive transfer of autoantibodies from mother to fetus is widely accepted. Clinical and experimental data support the pivotal role of the anti-SSA/Ro antibodies (directed against the 52 kDa or the 60kDa protein) in the development of autoimmune CHB. Indeed, in our systematic literature review, anti-SSA was by far the most frequently detected autoantibody in maternal sera (78.4% [295/376] of mothers). The pathogenetic roles of other autoantibodies, mainly anti-SSB/La and anti-U1RNP, are less clear and further obscured by the fact that, in most circumstances, these autoantibodies coexist with anti-SSA/Ro (in our review, only 15, 25, and 1 patient were positive only for anti-SSB, only ANA positivity, and anti-U1RNP, respectively). In mothers with SLE, ANA trans passes from placenta and works with anti SS-A/Ro to make harm in fetal thyroid tissue and myocardium[1]. Clinicians should consider testing for maternal anti-U1RNP and only ANA positivity in congenital heart block cases in which anti-SSA/Ro and/or anti-SSB/La are negative and there is no structural etiology [14].

More intriguing is the fact that 10.6% (40/376) of mothers tested were negative for any circulating autoantibody. The possibility of inter-laboratory differences in sensitivity must naturally be taken into account. Moreover, in a recent review, Brito-Zeron et al questioned the existence of “immunonegative” CHB, owing to the fact that most of such cases were diagnosed in children in adolescence or mothers who had not been tested for the full panel of potentially cardio toxic autoantibodies [15]*. *A maternal autoimmune disease is not a prerequisite for the development of NLE. On the contrary, our analysis shows that almost 32% of patients did not suffer from any disease at the time of birth of a child with NLE. Clearly, absence of maternal disease makes the diagnosis of NLE even more challenging because asymptomatic women will usually not be tested for autoantibodies. This highlights the need to test for the latter in all women of reproductive age who present to rheumatology care even for mild, nonspecific symptoms.

Our review attempted to capture all data regarding CHB subtypes and detection time in neonates born with NLE. Transplacental transfer of IgG antibodies from mother to fetus starts at 12th gestational week [3,4]. The period traditionally considered to confer the highest risk is during the middle to late parts of the second trimester, especially between the 16th and 26th weeks [3,4]. Indeed, we found that almost ¾ of cases (72.9%) were diagnosed between the 18th and 26th week of gestation, with a mean gestational age of ~ 24 weeks. However, we also found 4 cases (2.2%) diagnosed at weeks 16-17 and an additional 11.8% (n = 22), in which CHB was detected after 30th week of gestation, spanning a total period of more than 15 gestational weeks. Withstanding the fact that the majority of infants (78.0%) develop type III (i.e. complete) atrioventricular block, the rarity and of its occurrence as well as inability to predict, and the questionable efficacy -at best- of any therapeutic modality, we believe it may not be feasible to recommend strict weekly scheduling of foetal echocardiography throughout this whole period. Rather, we tend to favour a more practical approach regarding frequency of foetal echocardiography in pregnant women with circulating anti-SSA and/or anti-SSB antibodies, which consists of biweekly examinations for the period of 16–28 th gestational weeks [2]. This recommendation should by no means serve as a universal guide, but may provide a framework to reduce unnecessary stress on future mothers.

In our study we found that anti SSA/Ro antibody positivity was 84.6% and anti-SSB/La antibody positivity was 58.9% in cutaneous manifestations. On the other hand, anti U1-RNP antibody is responsible from skin involvement [14]. In our study, anti U1-RNP positivity was 11.5%.

Apart from cardiac involvement, involvement of other organs in the NLE has been much less characterized to date. Outside the skin, we found that hematologic involvement, hepatic dysfunction, pulmonary and neurologic complications, all appear in decreasing order of frequency in neonates with the NLE syndrome (15.5%, 10.3%, 1.3%, and 1.2%). Interestingly, pulmonary manifestations showed a predilection for male gender (62.5% of affected neonates were males), in contrast to the other system manifestations. Skin lesions, hematologic involvement, hepatobiliary involvement spontaneously disappear by cleaning of antibodies from the circulation of the baby at 6th to 12th months [16,2]. We were not able to characterize further the type of manifestations in each of these organ systems, as most of this information was not provided by original studies.

In 2 studies, recurrence of CHB was seen in 17% of pregnancies after the birth of a child with CHB [17,18]. Recurrence after first pregnancy was found as 12.1% in another study and all these recurrences were seen in patients with autoantibody (anti-SSA/Ro antibody, and anti-SSB/La) positivity, none of them were autoantibody negative [19]. In our study, none of the mothers with recurrent CHB baby were autoantibody negative. Additionally, percentage of recurrent pregnancy with NLE was 16.4%, CHB was 12.4%, and rash was 4%.

Certain limitations of our study deserve acknowledgment. First, due to strict methodologic limitations, we included only published studies which reported data on an individual patient basis. Inevitably, this has led to noninclusion of studies reporting on groups of patients, as these data could not be similarly analysed. Moreover, studies included in our systematic review had their own limitations, mainly in terms of missing data. Nevertheless, by including more than 750 patients with available detailed data, our literature review captured most clinical aspects of NLE, as well as intriguing phenotypic differences by race and ethnicity.

In conclusion, we performed a systematic literature review to portray the full clinical spectrum of NLE, beyond its most devastating consequence, autoimmune CHB. The latter is rather rare, and the possibility of its occurrence spans a substantial period within gestation. This, together with the paucity of therapeutic options (which we attempt to summarize in a separate systematic review), warrants a rational screening approach for its detection.

## Acknowledgement/Disclaimers/Conflict of Interest

All authors disclose no conflict of interest that may have influenced either the conduct or the presentation of the research. This work was supported by the Research Fund of the Hacettepe University, project number: TBB-2016-12742.

Abdulsamet Erden, Antonis Fanouriakis, Levent Kılıç, Alper Sarı, Berkan Armağan, Emre Bilgin, Yusuf Ziya Şener, Benazir Hymabaccus, Fatih Gürler, Serdar Ceylan participated in data collection, analyses, and writing of the manuscript; Sedat Kiraz, Ömer Karadağ, Dimitrios Boumpas participated in data analyses and writing of manuscript.
